# Integrated Analysis of Long Non-Coding RNAs and mRNAs Reveals Key Trans-Target Genes Associated with Heat Stress Response in *Rhododendron delavayi*

**DOI:** 10.3390/life15050697

**Published:** 2025-04-25

**Authors:** Changming Liu, Yang Liu, Xinyue Zhang, Lujie An, Zhiguo Tian

**Affiliations:** 1 College of Biomedical and Food Engineering, Shangluo University, Shangluo 726000, China; liuchangming@slxy.edu.cn (C.L.); l18291694055@outlook.com (Y.L.); zxy18391709349@outlook.com (X.Z.); alj18992985622@outlook.com (L.A.); 2 College of Art, Changzhou University, Changzhou 213164, China

**Keywords:** *R. delavayi*, lncRNA cis-acting, lncRNA trans-acting, lncRNAs, heat stress response

## Abstract

Long non-coding RNAs (lncRNAs) have been defined as non-coding transcripts exceeding 200 nucleotides, which play essential roles during transcriptional and post-transcriptional regulation in multiple biological processes. Understanding *R. delavayi* lncRNAs is of great significance as it is profoundly influenced by heat stress. In this research, a total of 1145 differentially expressed lncRNAs (DE-lncRNAs) and 9447 differentially expressed genes (DEGs) (log_2_|FC| > 1, *p* < 0.05) were characterized between heat-stress-treated groups and control groups. Further analysis showed that 229 DE-lncRNAs (144 upregulated and 85 downregulated) were commonly distributed in two comparisons (CK_vs._H3 and CK_vs._H6). We further investigated the cis- and trans-acting genes of the upregulated DE-lncRNAs, and found that 142 upregulated DE-lncRNAs corresponded to 1565 cis-acting DEGs, and 143 upregulated DE-lncRNAs corresponded to 3417 trans-acting DEGs. KEGG enrichment analysis of these target genes revealed that cis- and trans-acting DEGs of upregulated DE-lncRNA were primarily enriched in five and twelve KEGG pathways, respectively. Co-expression network analysis of upregulated DE-lncRNAs and DEGs enriched in the common KEGG pathways revealed 57 co-expression relationships between 28 DE-lncRNAs and 43 cis-acting DEGs and 554 co-expression relationships between 26 DE-lncRNAs and 90 trans-acting DEGs. Six DE-lncRNAs and six of their target DEGs were used as candidate genes to verify the RNA-seq data using qRT-PCR. Further analysis revealed three target genes (*TrxG*, *PEPC*, and *CCR*) out of six candidate DEGs that were selected as candidate genes for subsequent research. This study examined the relationship between DE-lncRNAs and DEGs and further screened out candidate DE-lncRNAs that can potentially provide an important theoretical basis and experimental data for the genetic improvement of heat tolerance in *R. delavayi*.

## 1. Introduction

Plants, as sessile organisms, have to continuously cope with various environmental stressors, including multiple biotic stresses (e.g., herbivore pests and pathogen diseases) and abiotic stresses (e.g., heavy metals, salinity, drought, and extreme temperature) [1,2,3]. Abiotic stress can give rise to cellular disorder and secondary stresses, such as oxidative and osmotic stress, protein denaturation, and membrane injury at the cellular level [2,4,5]. Heat stress is considered one of the most damaging abiotic stresses, impeding the normal growth and development of plants affecting and affecting global crop productivity [6]. LncRNAs, as a new frontier, play biological roles in regulating diverse cellular processes [7,8]. Most of the plant genome is transcribed largely as non-coding RNAs (ncRNAs), including tens of thousands of lncRNAs, whereas only ~2% encodes mRNAs based on high-throughput sequencing analysis, further indicating that lncRNAs may play essential roles in both the phenotype and adaptations of plants [8,9].

LncRNAs, which have been considered to lack protein-coding potential, have emerged as integral gene-expression regulators that underlie plant physiological and biochemical processes, as well as responses to various stresses [9,10]. Numerous studies have reported that lncRNAs are involved in regulating multiple processes of vegetative propagation and reproductive growth [7,8,10]. LncRNAs also play critical roles in mediating various environmental stress responses, including responses to extreme temperatures, heavy metals, drought, and salinity [7,8]. For example, overexpression of the *Arabidopsis* drought-induced lncRNA (DRIR) enhanced drought tolerance in transgenic plants compared with the control [11]. Quan et al. (2020) identified two Cd-responsive lncRNA gene pairs (MSTRG.22608-PtoMYB73 and MSTRG.5634-PtoMYB27) [12]. In *Betula platyphylla*, BplncSIR1 conferred salt tolerance by regulating *BpNAC2* to mediate reactive oxygen species scavenging and stomatal movement [13]. A total of 126 and 133 lncRNAs were identified in the M-81E and Roma lines of *Sorghum bicolor* under salt stress, respectively [14]. Moreover, the identification of 1229 DE-lncRNAs responsive to heat stress in *Brassica rapa* and the co-expression network revealed that these lncRNAs connected to heat shock proteins (HSPs) were involved in regulating heat stress response [15]. In heat-stress-treated *Populus qiongdaoensis* seedlings, it was identified that 25 DE-lncRNAs and one lncRNA (lncHSP18.2) could *cis*-regulate the gene expression of *HSP18.2* [16]. In pear, HILinc1 induced by heat stress increased the expression level of the PbHILT1 target gene by complementary base pairing, and PbHILT1 interacted with PbHSFA1 to enhance the expression level of PbMBF1c [17]. Plants overexpressing HILinc1 were therefore highly thermotolerant in the presence of heat stress [17]. However, the molecular mechanism of lncRNAs in *R. delavayi* under heat stress conditions is still unclear.

LncRNAs participate in regulating gene expression via a variety of mechanisms, having an impact on both proximal genes (cis-acting) and distal genes (trans-acting). The interaction between cis-acting lncRNAs and transcription factors/chromatin architecture precisely regulates various epigenetic processes (e.g., histone modification and chromatin loop), thereby influencing the transcript abundance of adjacent genes. In contrast, trans-acting lncRNAs serve as scaffolds for chromatin modifiers and transcriptional machinery, thereby regulating the transcript abundance of distantly located genes. This action also brings about alterations in protein function, stability, and localization. *R. delavayi* is an evergreen shrub with large scarlet flowers that make it highly attractive as an ornamental species [18,19]. The chromosome-level assembly of the *R. delavayi* genome makes it a valuable resource for genome-wide identification of lncRNAs, including determining the lncRNA’s location and boundaries, predicting structure and function, and revealing evolutionary characteristics [18]. Heat stress has adverse effects on *R. delavayi*, such as scorching and sunburning of its leaves and stems, resulting in the leaves turning yellow and dying [20]. However, the roles of lncRNAs in *R. delavayi* under heat stress conditions have not been reported. In the present study, several essential questions remain unexplored: Do lncRNAs participate in regulating the heat stress response of *R. delavayi*? Which lncRNAs are identified as key regulators involved in regulating heat stress response in *R. delavayi*? What is the difference among the various lncRNAs involved in the heat stress response of *R. delavayi*? Which target gene is regulated by key lncRNAs? The transcriptome sequencing (RNA-seq) helps to identify and characterize the lncRNAs and their corresponding target genes of key lncRNAs, which can help us to answer these questions [20].

In this study, a comparative transcriptome analysis of heat-stress-treated and non-treated *R. delavayi* plants was conducted to characterize the DE-lncRNAs and DEGs. Co-expression networks were applied for exploring the cis- and trans-acting genes of heat-stress-induced DE-lncRNAs. We further investigated the functions of cis- and trans-acting genes based on GO and KEGG enrichment analyses. Six DE-lncRNAs corresponding with five cis-acting genes and six DE-lncRNAs corresponding with three trans-acting genes were selected as candidates to verify the reliability of the RNA-seq results using qRT-PCR. This study provides a theoretical foundation for further functional studies of the candidate DE-lncRNAs and their corresponding targeted genes for improving the heat stress tolerance of *R. delavayi* using genetic modification techniques.

## 2. Materials and Methods

### 2.1. Plant Materials and Heat Stress Treatment

In this research, three-year-old *R. delavayi* seedlings grown in an experimental field in the south campus of Guizhou University were transferred into a climate chamber with 22 °C/18 °C (day/night) temperature, a photoperiod of 12/12 h (day/night), a relative humidity of 75%, and a light intensity of 1100 μmol m^−2^ s^−1^. After one month, 24 *R. delavayi* plants were transferred into an incubator to perform heat shock treatment at 40 °C for 3 days and 6 days. The non-treated and heat-stress-treated *R. delavayi* leaves were quickly sampled and frozen in liquid nitrogen and then stored at −80 °C for whole transcriptome sequencing and quantitative real-time reverse-transcription PCR (qRT-PCR) analysis.

### 2.2. Total RNA Extraction and Quality Examination

Total RNA was isolated from nine *R. delavayi* leaf samples under heat stress treatment using Trizol based on the manufacturer’s instructions. The NanoDrop and kaiaoK5500^®^ spectrophotometers were used to measure the concentrations and purity of total RNA, respectively. The RNA integrity of all samples was determined using an Agilent Bioanalyzer 2100 (Agilent Technologies, Diegem, Belgium) with an Agilent RNA 6000 kit (Agilent Technologies). The samples were sent to Baimaike Biotechnology Co., Ltd. (Beijing, China) for cDNA library construction and RNA sequencing. The remaining RNAs were stored at −80 °C for performing RT-qPCR analysis of candidate De-lncRNAs and their corresponding target genes.

### 2.3. Library Construction for RNA-Seq

Poly-T oligo-attached magnetic beads were applied for isolating mRNA from the total RNA. The 120~210 bp fragments of mRNA were generated using divalent cations under an elevated temperature, which were applied to construct libraries using an RNA library preparation kit based on strand Illumina instructions. The library construction was performed by Beijing Baimaike Biotechnology Co., Ltd. (Beijing, China), and sequencing was performed on the Illumina HiSeq X Ten system.

### 2.4. RNA-Seq Data Analysis

The clean reads were retrieved after filtering adapters and reads containing poly-N sequences, which were mapped in the *R. delavayi* genome obtained from the Ericaceae Genome Resource Database (TEGR) using HISAT2 (v2.0.5) software. After the alignment, Samtools (v.1.9) software was applied for sorting SAM files into BAM files, which were used as inputs to assemble the transcripts using Stringtie (v.1.3.5) software. All transcripts were merged to generate the final transcripts using cuffmerge [21].

According to the structural and functional characteristics of lncRNAs, we applied a range of screening procedures to identify the lncRNAs through the following five steps: (1) mono-exon transcripts were canceled; (2) the transcripts of ≤200 nt were filtered out; (3) transcripts overlapping protein-coding genes in sense orientation were removed; (4) transcripts with FPKM less than 0.5 were deleted; and (5) transcripts with coding potential as assessed by Coding Potential Calculator (Cpc) and the protein family database (Pfam). After the aforementioned steps, the remaining non-coding transcripts were considered as candidate lncRNAs, which were divided into two types (lincRNA and antisense lncRNA) through Cuffcompare.

### 2.5. Analysis of DE-lncRNAs and DEGs

StringTie software was applied to analyze the transcript abundance of both lncRNAs and mRNAs according to the Fragments Per Kilobase of transcript per Million mapped reads (FPKM) method. The DE-lncRNAs and DEGs were obtained using R software (version 3.2.3) [21]. DE-lncRNAs and DEGs with statistical significance between the heat-stress-treated group and the control group were characterized using volcano plot filtering with a fold-change threshold > 2 and *p* < 0.05. TBtools software was used to generate the heatmap and Venn diagrams of the differentially expressed transcripts [22].

### 2.6. Function Annotation and Co-Expression Network Construction

The heat-induced DE-lncRNAs were used to identify the potential trans- and cis-acting genes. In brief, the genes located within 100 kb up- or down-stream of the DE-lncRNAs were recorded as cis-acting genes. Pearson’s correlation coefficient (PCC) was applied to perform the correlation analysis between DE-lncRNAs and DEGs in samples at three different treatment stages [23]. The lncRNA-mRNA pairs with a |PCC| greater than 0.95 and a *p*-value less than 0.01 were treated as co-expression gene pairs, and the DEGs were considered as trans-acting target genes of lncRNAs.

To investigate the potential roles of DE-lncRNAs, the trans- and cis-acting genes of upregulated DE-lncRNAs in heat-stress-treated *R. delavayi* were used to perform GO (Gene Ontology) and KEGG (Kyoto Encyclopedia of Genes and Genomes) enrichment analyses using the GOseq R package and KOBAS software, respectively [24]. Gephi (v0.8.2) software was used to build the co-expression network of DE-lncRNAs and DEGs to screen the essential DE-lncRNAs involved in heat stress response [25].

### 2.7. qRT-PCR Analysis of Candidate DE-lncRNAs and DEGs

The mRNA was reverse-transcribed to the first-strand cDNA using QuantiTect^®^ Reverse Transcription kit (Qiagen, Hilden, Germany). Three biological and technical replicates were used in the RT-qPCR analysis using the Applied Biosystems 7500 Real-Time PCR system (Applied Biosystems, Waltham, MA, USA). The primers of DE-lncRNAs and DEGs candidates were designed and are listed in Table S1, and the *18S rRNA* gene encoding 18 S ribosomal RNA was considered as a reference gene (Table S1). The 2^−∆∆CT^ method was applied for analyzing the relative expression of DE-lncRNA and DEG candidates [26].

### 2.8. Statistical Analysis

SPSS-Statistical Package (version 5.0, IBM, Armonk, NY, USA) was used to perform statistical analyses. Analyses of variance (ANOVA) and mean separation were performed using t-tests and one-way ANOVA with the least significant difference (LSD) at *p* < 0.05.

## 3. Results

### 3.1. Comprehensive Overview of Transcriptome Sequencing (RNA-Seq)

RNA-seq was performed on nine *R. delavayi* leaf samples using the Illumina Hiseq Xten platform in this study. After filtering out the low-quality reads and adapters, a total of 91.29 Gb of clean bases distributed in 613,138,088 clean reads ranging from 60,341,510 to 75,965,538 for each library was obtained with a Q30 percentage > 92.24% and a GC content > 44.15% for the libraries. Moreover, 89.30–92.77% of clean reads were mapped into the *R. delavayi* genome for each library. These results further demonstrate that RNA-seq data are of high quality and suitable for subsequent analyses (Table 1).

### 3.2. Identification of lncRNAs and mRNA in R. delavayi Under Heat Stress Treatment

**Figure 1:**
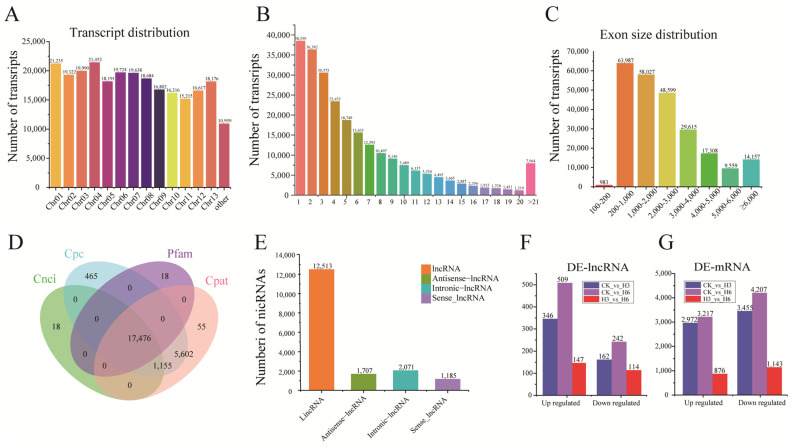
Identification of lncRNAs associated with heat stress response of *R. delavayi.* (**A**) The distribution of identified transcripts across *R. delavayi* chromosomes. The X axis represents the number of transcripts, and the Y axis represents the different chromosomes. (**B**) Exon numbers for each transcript of lncRNAs in *R. delavayi* under heat stress treatment. The X axis represents the number of transcripts, and the Y axis represents the number of exons. (**C**) The distribution of exon length of the identified transcripts. The X axis represents the number of transcripts, and the Y axis represents the exon size. (**D**) Venn diagram of lncRNAs with coding potential. Cpc: Coding Potential Calculator; Cnci: Coding-Non-Coding Index; Pfam: the protein family database; Cpat: Coding-Potential Assessment Tool. (**E**) The classification of lncRNAs. The X axis represents the number of lncRNAs, and the Y axis represents the different types of lncRNAs. (**F**) Identification of DE-lncRNA and DEGs in the comparison of heat-stress-treated groups and control groups. The dark blue, purple, and red column charts represent the number of differentially expressed lncRNAs in CK vs. H3, CK vs. H6, and H3 vs. H6, respectively. (**G**) Identification of DEGs in the comparison of heat-stress-treated groups and control groups. The dark blue, purple, and red column charts represent the number of differentially expressed genes in CK vs. H3, CK vs. H6, and H3 vs. H6, respectively.

To investigate the molecular regulatory mechanism of lncRNAs in the *R. delavayi* heat stress response, we sought to identify and analyze lncRNAs in *R. delavayi* that had been exposed to short-term heat stress. A total of 50,815 transcripts were detected in heat-stress-treated *R. delavayi*, and all transcripts were unevenly distributed among 13 *R. delavayi* chromosomes. Among them, chromosome 04 contained the largest number of transcripts (21,452, 8.42%), while chromosome 11 contained the smallest number of transcripts (15,215), accounting for 6.03% of the total transcripts (Figure 1A). A total of 15.91% of the transcripts (38,539) had only one exon, whereas 3.29% of the transcripts (7964) had more than 20 exons (Figure 1B). Moreover, the length distribution of all transcripts was also investigated. It was found that the length distributions of lncRNAs ranged from 202 to 11,603 nt, with 12,621 (72.22%), 3231 (18.49%), 1107 (6.33%), and 519 (2.99%) processed with lengths of 200–1000, 1000–2000 nt, 2000–3000 nt, and >3000 nt, respectively (Figure 1C). Four tools, including the Coding Potential Calculator (CPC), Pfam-scan (PFAM), Coding–Non-Coding Index (CNCI), and Coding Potential Assessing Tool (CPAT), were applied to characterize the potential of the lncRNAs. A total of 17,476 non-coding transcripts shared in the intersection among CPC, PFAM, CNCI, and CPAT are shown in the Venn diagram in Figure 1D. We compared these lncRNAs with those collected in several specific databases and found that 153 transcripts were processed with a NONCODE transcript ID, and 17,323 transcripts had no transcript IDs (NA), suggesting that these lncRNAs (17,323) without transcript IDs are novel lncRNAs. The identified lncRNAs were each assigned a Cufflinks class code, including 12,513 lincRNAs (71.6%), 1707 antisense lncRNAs (9.8%), 2071 intronic lncRNAs (11.9%), and 1185 sense lncRNAs (Figure 1E).

### 3.3. Identification of DE-lncRNAs and DEGs

**Figure 2:**
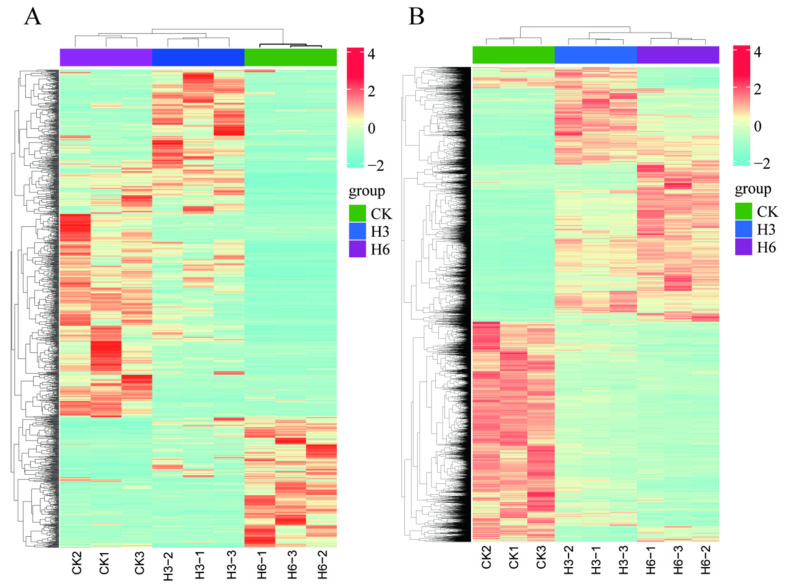
Expression patterns of DE-lncRNAs and DEGs. (**A**) Expression patterns of DE-lncRNAs in the comparison of heat-stress-treated groups and the control group. (**B**) Expression patterns of DEGs in the comparison of heat-stress-treated groups and the control group. Red and green boxes represent the up- and downregulated lncRNAs (**A**) and genes (**B**).

The transcripts exhibiting log2|FC| > 1 and *p* < 0.05 were used as criteria to identify DE-lncRNAs and DEGs. We fully characterized 1145 DE-lncRNAs and 9447 DEGs between the heat-stress-treated group and the control group. In the CK_vs._H3 comparison group, a total of 508 DE-lncRNA transcripts (346 upregulated and 162 downregulated) were differentially expressed, and 751 DE-lncRNAs (509 upregulated and 242 downregulated) were in the comparison of CK_vs._H6 (Figure 1E). Further analysis found that 229 DE-lncRNAs were commonly distributed in two comparisons (CK_vs._H3 and CK_vs._H6). Moreover, we identified 6427 DEGs (2972 upregulated and 3455 downregulated) in the comparison of CK_vs._H3, 7424 DEGs (3217 upregulated and 4207 downregulated) in the comparison of CK_vs._H6, and 2019 DEGs (876 upregulated and 1143 downregulated) in the comparison of H3_vs._H6 (Figure 1D). Moreover, a heat map of the expression of DE-lncRNA and DEGs was generated, and the results of the cluster analysis demonstrated significant differences in the expression patterns of DE-lncRNA and DEGs between the heat-stress-treated groups and the control group (Figure 2A,B, Tables S2 and S3).

Further analysis revealed that 229 DE-lncRNAs (144 upregulated and 85 downregulated) were used to conduct trans- and cis-target gene prediction through log_2_ |FC| > 1 and *p* < 0.05 parameters. For predicting cis-acting DEGs of upregulated DE-lncRNAs, the genes distributed in 100 kb up- and down-stream of DE-lncRNAs were applied to identify the cis-target genes of 144 DE-lncRNAs. Our results reveal that 142 DE-lncRNAs corresponded to 1565 cis-acting DEGs (Table S4). To predict the trans-target genes of 144 DE-lncRNAs, the Spearman’s correlation test was applied to explore the co-expression relationship between DE-lncRNAs and trans-acting DEGs. The results reveal that the interactions of DE-lncRNAs and DEGs with R values ≥ 0.9 and *p* < 0.05 were used as strands for identifying 3417 trans-acting DEGs in 143 DE-lncRNAs (Table S5).

### 3.4. GO and KEGG Enrichment Analysis

**Figure 3:**
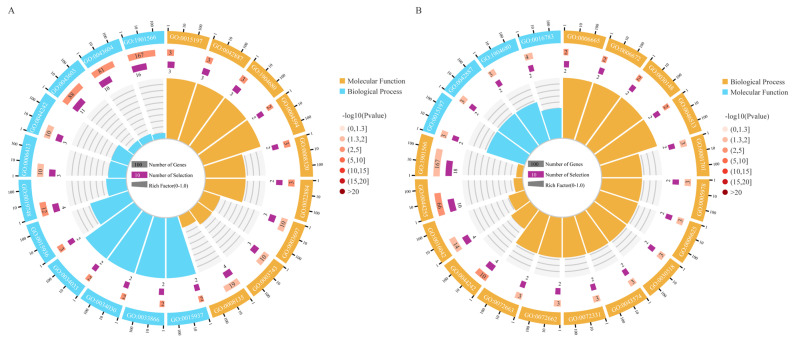
GO analysis of target DEGs of upregulated DE-lncRNAs. (**A**) GO analysis of cis-acting DEGs of upregulated DE-lncRNAs. (**B**) GO analysis of trans-acting DEGs of upregulated DE-lncRNAs.

GO analysis was used to analyze the functions of cis- and trans-target genes of upregulated DE-lncRNAs by determining their similarity with other genes of known functions. In this study, GO analyses of cis- and trans-target genes of 144 DE-lncRNAs upregulated in heat-stress-treated *R. delavayi* were performed. The results of the GO analyses revealed that cis-acting DEGs were mostly enriched in molecular functions (e.g., peptide transporter activity, amide transmembrane transporter activity, translation factor activity, RNA binding, and peptide transmembrane transporter activity) and biological processes (e.g., the secondary metabolic process, the cellular lipid catabolic process, the amide biosynthetic process, and the coenzyme A metabolic process) (Table S6). Similarly, the trans-target genes were mostly involved in molecular functions (peptide transporter activity, amide transmembrane transporter activity, peptide transmembrane transporter activity, and sulfurtransferase activity) and biological processes (e.g., the sphingolipid metabolic process, the glycogen biosynthetic process, the intracellular steroid hormone receptor signaling pathway, the cellular lipid catabolic process, the lipid metabolic process, and the organonitrogen compound biosynthetic process) (Figure 3 and Table S7). These results indicate a variation in the transcriptome between the two groups.

**Figure 4:**
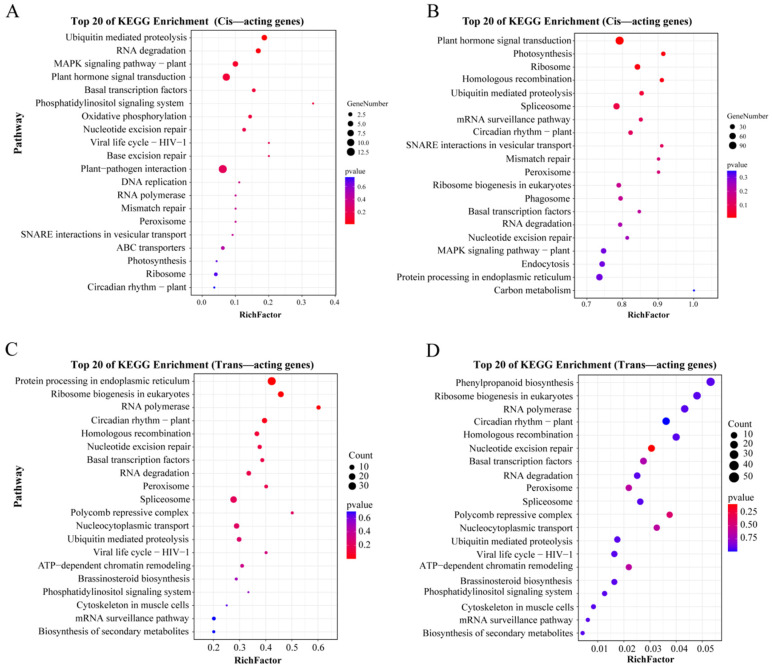
KEGG enrichment analysis of target genes of upregulated DE-lncRNAs in heat-stress-treated *R. delavayi*. (**A**) KEGG enrichment analysis of cis-acting DEGs in CK_vs._H3; (**B**) KEGG enrichment analysis of cis-acting DEGs in CK_vs._H6; (**C**) KEGG enrichment analysis of trans-acting DEGs in CK_vs._H3; (**D**) KEGG enrichment analysis of trans-acting DEGs in CK_vs._H6. KEGG enrichment analyses were arranged in ascending order of *p* value, and the top 20 objects were selected successively.

KEGG enrichment analyses of target genes of upregulated DE-lncRNAs were conducted to analyze whether the target genes were statistically enriched in some biological pathways involved in heat stress response. In the present study, KEGG enrichment analyses of cis-acting DEGs in CK_vs._H3 and CK_vs._H6 were conducted, respectively (Figure 4). The top 20 pathways were selected based on the EnrichR combined score. Five significant enrichment pathways were commonly distributed in two pairwise comparison groups (CK_vs._H3 and CK_vs._H6), including diterpenoid biosynthesis (four cis-acting DEGs), RNA polymerase (eight cis-acting DEGs), sphingolipid metabolism (eight cis-acting DEGs), phenylpropanoid biosynthesis (eighteen cis-acting DEGs), and glutathione metabolism (sixteen cis-acting DEGs) (Figure 4A,B, and Supplementary Tables S8 and S9). Moreover, KEGG enrichment analyses were performed for trans-acting genes differentially expressed in CK_vs._H3 and CK_vs._H6. Among the top 20 pathways, twelve significant enrichment pathways were detected in two pairwise comparison groups, including alanine, aspartate, and glutamate metabolism (52 trans-acting DEGs); lysine degradation (22 trans-acting DEGs); carbon metabolism (237 trans-acting DEGs); biosynthesis of amino acids (214 trans-acting DEGs); glycolysis/gluconeogenesis (137 trans-acting DEGs); RNA degradation (124 trans-acting DEGs); beta-alanine metabolism (47 trans-acting DEGs); glycine, serine, and threonine metabolism (66 trans-acting DEGs); carbon fixation in photosynthetic organisms (61 trans-acting DEGs); linoleic acid metabolism (23 trans-acting DEGs); riboflavin metabolism (22 trans-acting DEGs); and arginine and proline metabolism (8 trans-acting DEGs) (Figure 4C,D, and Supplementary Tables S10 and S11). These results indicate that these pathways might play important roles in regulating the heat stress response in *R. delavayi*.

### 3.5. Co-Expression Networks of lncRNAs and Their Cis and Trans-Target Genes

**Figure 5:**
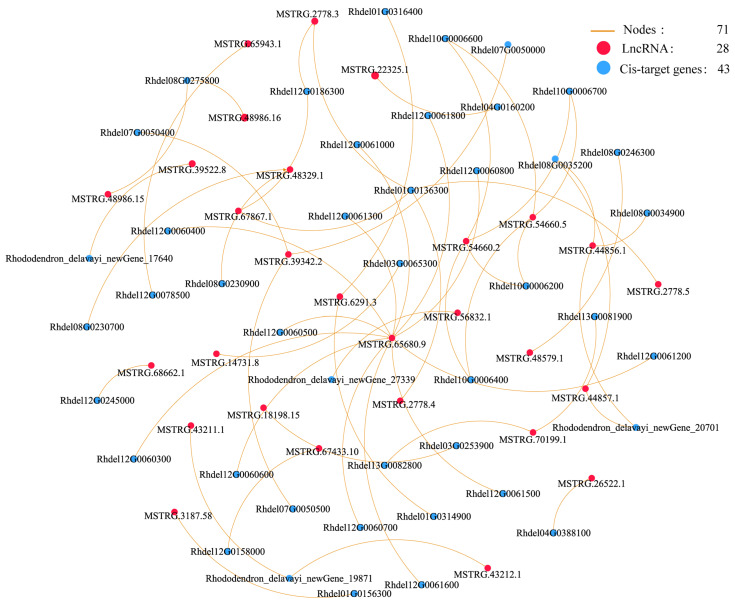
Co-expression network of De-lncRNAs and their corresponding cis-acting DEGs enriched in five pathways. The orange line, solid red circle, and solid blue circle represent interaction nodes, DE-lncRNAs, and cis-acting DEGs, respectively.

**Figure 6:**
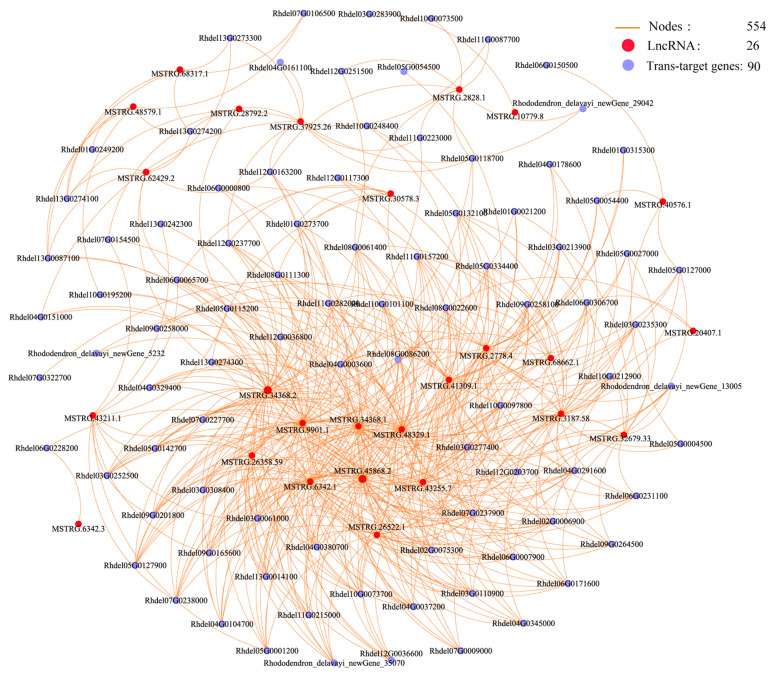
Co-expression network of De-lncRNAs and their corresponding trans-acting genes enriched in twelve pathways. The orange line, solid red circle, and solid blue circle represent represent interaction nodes, DE-lncRNAs, and trans-acting genes, respectively.

To investigate the potential roles of lncRNAs, the co-expression networks of DE-lncRNA and target gene co-occurring pathways were constructed using Cytoscape software (v. 3.5.1). The interaction network of De-lncRNAs and their corresponding cis-acting DEGs enriched in five pathways was constructed. The results reveal 128 interaction nodes, including 28 upregulated De-lncRNAs and 43 cis-acting DEGs. These nodes formed 57 network pairs (Figure 5 and Table S12). We also constructed the co-expression network of DE-lncRNAs and their corresponding trans-acting genes enriched in twelve pathways comprising 554 nodes, including 26 DE-lncRNAs and 90 trans-acting DEGs (Figure 6 and Table S13). Co-expression networks revealed that a single DE-lncRNA could interact with multiple cis- and trans-acting genes. For example, the MSTRG.65680.9 was associated with 12 cis-acting DEGs, MSTRG.48329.1 interacted with 4 cis-acting DEGs, and MSTRG.54660.2 connected to 4 cis-acting DEGs. The MSTRG.34368.2 was associated with 41 trans-target DEGs, the MSTRG.2778.4 was associated with 31 trans-acting DEGs, and the MSTRG.34368.1 was associated with 38 trans-acting DEGs.

### 3.6. RT-qPCR Validation

**Figure 7:**
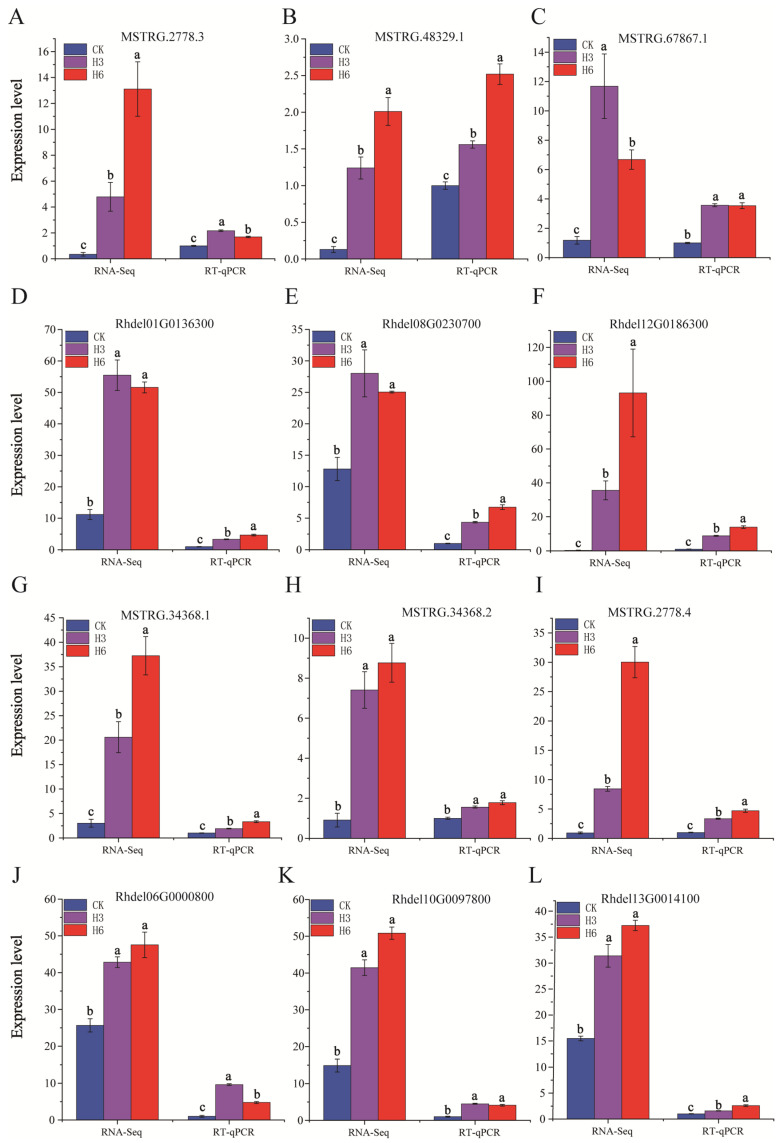
The qRT-PCR validation of DE-lncRNAs and cis-acting DEGs. (**A**–**C**,**G**–**I**) Investigation of the expression patterns of six upregulated DE-lncRNAs using qRT-PCR. (**D**–**F**,**J**–**L**) Investigation of the expression patterns of six target DEGs using qRT-PCR. The transcript abundance of six DE-lncRNAs and five cis-acting DEGs relative to 18S rRNA determined by qRT-PCR. Three technical replicates and three biological replicates were applied for each data point. Data were presented as means ± SD (N = 6). Lower case letters indicate significant differences between heat-stress-treated group and CK group.

Based on the expression analysis and gene function annotation, three pairs of DE-lncRNAs and cis-regulated DEGs involved in heat stress response were screened, including MSTRG.2778.3-*PPABC1* (Rhdel01G0136300), MSTRG.67867.1-*UGT89B2* (Rhde112G0186300), and MSTRG.48329.1-*CCR* (Rhdel08G0230700). Moreover, three De-lncRNAs (MSTRG.34368.2, MSTRG.2778.4, and MSTRG.34368.1) connected to three trans-regulated DEGs, such as *TrxG* (Rhde106G0000800), *PEPC* (Rhde110G0097800), and *IDH* (Rhde113G0014100), were selected as candidates involved in regulating heat stress response (Table 2). To confirm the reliability of the RNA-seq results, six candidate DE-lncRNAs and their corresponding six target genes (three cis-acting and three trans-acting) enriched in the KEGG pathways related to the heat stress response of *R. delavayi* were selected for qRT-PCR validation. The results of the RT-qPCR of the three DE-lncRNAs and their corresponding three cis-acting DEGs reveal similar expression trends in comparison with the findings of the RNA-seq data (Figure 7), and the expression patterns of the three DE-lncRNAs and their three trans-acting DEGs are consistent with the results of the RNA-seq data. This result further confirms the accuracy and reproducibility of RNA-seq data, which indicates that these DE-lncRNAs and their corresponding target genes might be involved in the heat stress response in *R. delavayi*. Among them, the expression patterns of three target genes (*CCR*, *TrxG*, and *PEPC*) were consistent with the findings of previous studies in other plants, which were further selected as candidate genes for subsequent research.

## 4. Discussion

The genomes of plants and other eukaryotes are transcribed in a developmentally regulated manner, resulting in the generation of a substantial amount of non-coding RNA (ncRNA). These are involved in the interaction of other nucleic acids and proteins that have a wide influence on cell biology [27,28]. Long non-coding RNAs have been reported to participate in regulating various biological processes [8,9,10]. Previous studies have revealed that lncRNAs are involved in regulating plant growth and development, as well as in response to various environmental stressors [11,12,13,14]. Heat stress causes considerable damage to *R. delavayi*, including leaf scorching, sunburn on leaves and stems, and leaf senescence and abscission [20,29]. Although the roles of lncRNAs involved in regulating the response to various environmental stressors in some plants have been widely studied, the functions of lncRNAs in the heat stress response of *R. delavayi* were still unclear. In this study, RNA-seq was used to investigate the molecular regulatory mechanism of lncRNAs in the heat stress response of *R. delavayi.* The results reveal that a total of 17,476 lncRNAs (12,513 lincRNAs, 1707 antisense lncRNAs, 2071 intronic lncRNAs, and 1185 sense lncRNAs) and 50,649 mRNAs were identified as participants in the heat stress response of *R. delavayi.*

Further analysis identified 1145 DE-lncRNAs and 9447 DEGs that were differentially expressed in the heat-stress-treated group in comparison with the control groups, suggesting that lncRNAs play important roles in the heat stress response of *R. delavayi*. This result is similar to the results of previous studies. For example, He et al. (2019) revealed that a total of 108 lncRNAs and 2130 mRNAs were differentially expressed in cucumber leaves treated with high temperatures [30]. A total of 1614 lncRNAs and 25,665 mRNAs were differentially expressed in response to heat and drought stress in *B. juncea* [31]. LncRNAs often function as essential cis- and trans-target regulators for activating and inhibiting the expression of protein-coding genes [8,32,33]. Numerous studies have revealed that lncRNAs play important roles in the regulation of gene expression by both cis- and trans-regulatory mechanisms [32,33,34]. In this study, the cis- and trans-acting DEGs of 144 upregulated DE-lncRNAs in heat-stress-treated *R. delavayi* were investigated, and it was found that 141 DE-lncRNAs corresponded to 867 cis-acting DEGs and 126 DE-lncRNAs corresponded to 2098 trans-acting DEGs.

KEGG enrichment analysis of cis- and trans-acting genes of upregulated DE-lncRNAs revealed that cis-acting DEGs were significantly enriched in five pathways, such as diterpenoid biosynthesis, RNA polymerase, sphingolipid metabolism, phenylpropanoid biosynthesis, and glutathione metabolism. Our results were consistent with the findings of the previous studies [35,36]. Previous studies have revealed that these pathways may be involved in heat stress response in many plants [37,38,39]. In *Solanum tuberosum*, the diterpenoid biosynthesis and phenylpropanoid biosynthesis pathways were enriched in heat-stress-treated tubers based on the KEGG enrichment analysis [40]. In barley, male sterility was caused by the phosphorylation of serine-5 within the RNA polymerase II C-terminal domain, which changed the gene expression associated with early anther development under high-stress treatment [41]. Dard et al. (2023) revealed that glutathione was involved in mediating thermomorphogenesis and heat stress responses in *Arabidopsis thaliana* [42]. Moreover, KEGG enrichment analysis of trans-acting DEGs revealed twelve significant enrichment pathways, including alanine, aspartate, and glutamate metabolism; lysine degradation; carbon metabolism; the biosynthesis of amino acids; glycolysis/gluconeogenesis; RNA degradation; beta-alanine metabolism; glycine, serine, and threonine metabolism; carbon fixation in photosynthetic organisms; linoleic acid metabolism; riboflavin metabolism; and arginine/proline metabolism. Numerous studies have reported that these pathways are significantly influenced by heat stress [43,44]. Zheng et al. (2022) revealed that several amino acid metabolism pathways (alanine, aspartate, glutamate metabolism, beta-alanine, lysine degradation, glycine, serine and threonine metabolism, and arginine/proline) were all significantly regulated in jojoba leaves under heat stress [44]. Hewitt et al. (2023) [43] revealed that the effects of elevated temperature are expected to elicit a notable effect on glycolysis/gluconeogenesis. The above research further confirms our findings [43].

The combined co-expression network and KEGG enrichment analysis, three DE-lncRNAs and their corresponding three cis-acting DEGs (*POLR2L*, *CCR*, and *UGT89B2*), and three DE-lncRNAs and their corresponding three trans-acting genes (*TrxG*, *PEPC*, and *IDH*) were significantly increased in heat-stress-treated *R. delavayi* based on RNA-seq and qRT-PCR results, which is consistent with previous studies. RNA-seq and RT-qPCR analyses revealed the high transcriptional level of *PtrCCR2* (Potri.003G181400) in heat-stress-treated poplar, suggesting that this gene plays an essential role in increasing lignin content, and its metabolic intermediates were induced by high temperatures [45]. Moreover, six DE-lncRNAs and their corresponding three trans-acting genes (*TrxG*, *PEPC*, and *IDH*) were upregulated in heat-stress-treated *R. delavayi* based on RNA-seq and qRT-PCR analyses, which are consistent with the findings of previous studies. Song et al. (2020) revealed that the overexpression of the *ATrxG1* gene enhances the heat stress tolerance in transgenic *Arabidopsis* [46]. Qi et al. (2017) revealed that the *ZmPEPC* gene could improve antioxidant enzyme activity, increase the transcript abundance of photosynthesis-related genes, and inhibit chlorophyll degradation, which ultimately improves heat stress tolerance [47].

## 5. Conclusions

This research first investigated lncRNAs and their corresponding target genes associated with heat stress response in *R. delavayi.* The co-expression network of DE-lncRNAs and target genes revealed 142 upregulated DE-lncRNAs, corresponding with 1565 cis-acting DEGs, and 143 DE-lncRNAs, corresponding with 3417 trans-acting DEGs. KEGG enrichment analysis revealed that cis- and trans-acting DEGs, commonly distributed in two groups, were primarily enriched in five and twelve pathways, respectively. Combined with the function annotation of target genes, three DE-lncRNAs and their corresponding three cis-acting DEGs, and three DE-lncRNAs and their corresponding three trans-acting DEGs were used as candidate genes to verify the RNA-seq data using qRT-PCR. Further analysis revealed that three target genes (*TrxG*, *PEPC*, and *CCR*) were selected as candidate genes for further study. This study has examined the relationship between DE-lncRNAs and DEGs and further screened DE-lncRNA candidates that can potentially provide an important theoretical basis and experimental data for the genetic improvement of heat tolerance in *R. delavayi*.

## Figures and Tables

**Table 1:** Detailed information regarding the RNA-seq.

Sample	Clean Reads	Mapped Reads	Uniq Mapped Reads	Multiple Mapped Reads	GC (%)	Q30 (%)
CK1	72,796,176	65,816,206 (90.41%)	59,540,530 (81.79%)	6,275,676 (8.62%)	44.11	94.4
CK2	66,000,982	60,399,258 (91.51%)	54,745,987 (82.95%)	5,653,271 (8.57%)	44.45	94.69
CK3	72,965,128	67,690,520 (92.77%)	62,163,128 (85.20%)	5,527,392 (7.58%)	44.39	95.1
H3-1	61,128,826	54,812,099 (89.67%)	50,337,543 (82.35%)	4,474,556 (7.32%)	44.68	94.28
H3-2	63,427,652	56,640,331 (89.30%)	52,148,500 (82.22%)	4,491,831 (7.08%)	44.53	94.55
H3-3	70,936,282	63,721,989 (89.83%)	58,902,339 (83.04%)	4,819,650 (6.79%)	44.44	94.3
H6-1	60,341,510	54,375,726 (90.11%)	50,600,561 (83.86%)	3,775,165 (6.26%)	44.27	94.28
H6-2	75,965,538	68,870,447 (90.66%)	63,723,386 (83.88%)	5,147,061 (6.78%)	44.34	94.24
H6-3	69,575,994	62,557,432 (89.91%)	57,860,201 (83.16%)	4,697,231 (6.75%)	44.9	94.62

Note: Q30% represents the proportion of the data in which the quality values are >Q30 in the raw data. H3 and H6 represent *R. delavayi* plants under heat stress treatment at 38 °C for 3 days and 6 days. CK represents control. GC represents GC content.

**Table 2:** Prediction of gene function of candidate DE-lncRNAs and DEG candidates.

LncRNA	Target Genes	KEGG Pathways	Gene Annotation	Abbr.
MSTRG.2778.3	Rhdel01G0136300	RNA polymerase	RNA polymerase subunit	PPABC1
MSTRG.48329.1	Rhdel08G0230700	Phenylpropanoid synthesis	Cinnamoyl-CoA reductase 1	CCR
MSTRG.67867.1	Rhdel12G0186300	Phenylpropanoid synthesis	UDP-glucosyltransferas	UGT89B2
MSTRG.34368.2	Rhde106G0000800	Val, leu, Ile degradation	Trithorax-related proteins	TrxG
MSTRG.2778.4	Rhde110G0097800	Linoleic acid metabolism	Phosphoenolpyruvate carboxylase	PEPC
MSTRG.34368.1	Rhde113G0014100	Val, leu, Ile degradation	Isocitrate dehydrogenase	IDH

## Data Availability

RNA sequencing data were deposited at the National Center for Biotechnology Information (NCBI) in the Sequence Read Archive (SRA) under Bioproject accession number PRJNA1249916.

## Supplementary Materials

The following supporting information can be downloaded at: https://www.mdpi.com/article/10.3390/life15050697/s1. Figure S1: Sample clustering and principal component analysis (PCA) of differentially expressed transcripts. (A), Cluster analysis. (B), PCA. Table S1: The primers used in this study. Table S2: The expression patterns of DE-lncRNAs in heat-stress-treated *R. delavayi*. Table S3: The expression patterns of DEGs in heat-stress-treated *R. delavayi*. Table S4: The cis-acting DEGs of upregulated DE-lncRNAs. Table S5: The trans-acting DEGs of upregulated DE-lncRNAs. Table S6: GO analysis of cis-acting DEGs. Table S7: GO analysis of trans-acting DEGs. Table S8: KEGG enrichment analysis of cis-acting DEGs in the comparison CK vs. H3. Table S9: KEGG enrichment analysis of cis-acting DEGs in the comparison CK vs. H6. Table S10: KEGG enrichment analysis of trans-acting DEGs in the comparison CK vs. H3. Table S11: KEGG enrichment analysis of trans-acting DEGs in the comparison CK vs. H6. Table S12: The cis-acting DEGs commonly enriched KEGG pathways of 28 upregulated DE-lncRNAs. Table S13: The trans-acting DEGs commonly enriched KEGG pathways of 26 upregulated lncRNAs.
