# Supplementary material for: Integrated Analysis of Long Non-Coding RNAs and mRNAs Reveals Key Trans-Target Genes Associated with Heat Stress Response in Rhododendron delavayi
Source: Life (Basel). 2025 Apr 25;15(5):697. doi: 10.3390/life15050697 (PMC12113157; doi:10.3390/life15050697)
Supplement: Supplementary file 1 [file life-15-00697-s001.zip › Table S1.pdf]

Table S1. The primers used in this study

Gene name	Forward primer (5'to 3')	Reverse primer (5' to 3')
RhdelGene_27339	CTCCTACTACGGCGAAACAAG	GCGTAGAGCCTGTCGATAATG
Rhdel01G0136300	GACAGCCCTGACCAGATTTATG	CCCTAGCAAAGGGAGTTAGGT
Rhdel08G0230700	GCCAACTCTCAATCTCACTTCC	CTGCCACTAGCTGAAGAAACC
Rhdel12G0186300	GCTTGAGAGAACCCGAGTTAAG	CTCGACCAACTCGTCCAAATC
Rhdel02G0312200	GGCGTTCCAGATCATTGGTATC	CTACGGCTTGAACCAACGTAAG
Rhdel06G0000800	GTAGCTCCAGCCAACAGATAC	CCCTCATCGTCCTCAAAGAAC
Rhdel10G0097800	GACTACCGAGGATGACCTTTG	CACGAATACCTTGTCCAGAGAG
Rhdel13G0014100	CTGTTAGTGGTTCTGAGGATGG	CGTCCTTTCCTAGGGTTTCTTC
MSTRG.56832.1	CATCGGAAAGGTCCCAAGATAC	CCCTTTAGGGACATGTCTAGGA
MSTRG.2778.3	AGATCTCCTTCCTAGCCTCTTC	GCGTAGTATCTGCACTCAACC
MSTRG.48329.1	CGGACTGCGGAATAATCTCTC	GTCGAGACACCTACTCGTATCA
MSTRG.67867.1	CCGGCTCTCTTTCTCTCTCTATC	GGTGGGAATGAGCTTGATCTTG
MSTRG.13003.77	GTCGAGAGTTCCCGTGAAATAG	CCAGTGGATCCTACCAAAGTG
MSTRG.13013.1	GATAGAACGGGCTGTCGTGTT	GAATGTGAATCCACTGCCATGC
MSTRG.2778.4	GATCTCCTTCCAAGCCTCTTC	GCGTAGTATCTGCACTCAACC
MSTRG.4315.4	GTTTGGAACCCTTTGGTCGTAG	GAGCTACACAAGCCCAGTAGA
MSTRG.34368.1	GACGCTATTGGTTATGGGTCCT	CAGAGAGCAGTCAGTGTTCGAT
MSTRG.45868.2	CCGCTTGTGGGTTCCATAATAG	CCTCCAGGGAGGCACATAATA
MSTRG.34368.2	GACGCTATTGGTTATGGGTCCT	CAGAGAGCAGTCAGTGTTCGAT
MSTRG.48329.1	CTCCTTGTGCCCTAGCTGAATA	CCACAAAGAAATCCACCACCGA
18s	GGGGCATTCGTATTTCATAGTC	CGGTATCTGATCGTCTTCGAG
