# Supplementary material for: Integrated Analysis of Long Non-Coding RNAs and mRNAs Reveals Key Trans-Target Genes Associated with Heat Stress Response in Rhododendron delavayi
Source: Life (Basel). 2025 Apr 25;15(5):697. doi: 10.3390/life15050697 (PMC12113157; doi:10.3390/life15050697)
Supplement: Supplementary file 1 [file life-15-00697-s001.zip › Table S12.pdf]

Table S12 The cis-acting DEGs commonly enriched KEGG pathways of 28 upregulated DE-lncRNAs.

Source	Target	Source	Target
Rhdel03G0065300	MSTRG.14731.8	Rhdel10G0006600	MSTRG.54660.2
Rhdel03G0253900	MSTRG.18198.15	Rhdel10G0006700	MSTRG.54660.2
Rhdel04G0160200	MSTRG.22325.1	Rhdel10G0006200	MSTRG.54660.5
Rhdel04G0388100	MSTRG.26522.1	Rhdel10G0006400	MSTRG.54660.5
Rhdel01G0136300	MSTRG.2778.3	Rhdel10G0006600	MSTRG.54660.5
Rhdel12G0186300	MSTRG.2778.3	Rhdel10G0006700	MSTRG.54660.5
Rhdel01G0136300	MSTRG.2778.4	Rhododendron_delavayi_newGene_27339	MSTRG.56832.1
Rhdel01G0136300	MSTRG.2778.5	Rhdel01G0314900	MSTRG.6291.3
Rhdel01G0156300	MSTRG.3187.58	Rhdel01G0316400	MSTRG.6291.3
Rhdel07G0050000	MSTRG.39342.2	Rhdel12G0060300	MSTRG.65680.9
Rhdel07G0050400	MSTRG.39342.2	Rhdel12G0060400	MSTRG.65680.9
Rhdel07G0050500	MSTRG.39342.2	Rhdel12G0060500	MSTRG.65680.9
Rhododendron_delavayi_newGene_17640	MSTRG.39522.8	Rhdel12G0060600	MSTRG.65680.9
Rhododendron_delavayi_newGene_19871	MSTRG.43211.1	Rhdel12G0060700	MSTRG.65680.9
Rhododendron_delavayi_newGene_19871	MSTRG.43212.1	Rhdel12G0060800	MSTRG.65680.9
Rhdel08G0034900	MSTRG.44856.1	Rhdel12G0061000	MSTRG.65680.9
Rhdel08G0035200	MSTRG.44856.1	Rhdel12G0061200	MSTRG.65680.9
Rhododendron_delavayi_newGene_20701	MSTRG.44856.1	Rhdel12G0061300	MSTRG.65680.9
Rhdel08G0035200	MSTRG.44857.1	Rhdel12G0061500	MSTRG.65680.9
Rhododendron_delavayi_newGene_20701	MSTRG.44857.1	Rhdel12G0061600	MSTRG.65680.9
Rhdel08G0230700	MSTRG.48329.1	Rhdel12G0061800	MSTRG.65680.9
Rhdel08G0230700	MSTRG.48329.1	Rhdel12G0078500	MSTRG.65943.1

Rhdel08G0230900	MSTRG.48329.1	Rhdel12G0158000	MSTRG.67433.10
Rhdel08G0230700	MSTRG.48329.1	Rhdel12G0186300	MSTRG.67867.1
Rhdel08G0246300	MSTRG.48579.1	Rhdel01G0136300	MSTRG.67867.1
Rhdel08G0275800	MSTRG.48986.15	Rhdel12G0245000	MSTRG.68662.1
Rhdel08G0275800	MSTRG.48986.16	Rhdel13G0081900	MSTRG.70199.1
Rhdel10G0006200	MSTRG.54660.2	Rhdel13G0082800	MSTRG.70199.1
Rhdel10G0006400	MSTRG.54660.2		
