# Supplementary figures and images for: Integrated Analysis of Long Non-Coding RNAs and mRNAs Reveals Key Trans-Target Genes Associated with Heat Stress Response in Rhododendron delavayi
Source: Life (Basel). 2025 Apr 25;15(5):697. doi: 10.3390/life15050697 (PMC12113157; doi:10.3390/life15050697)

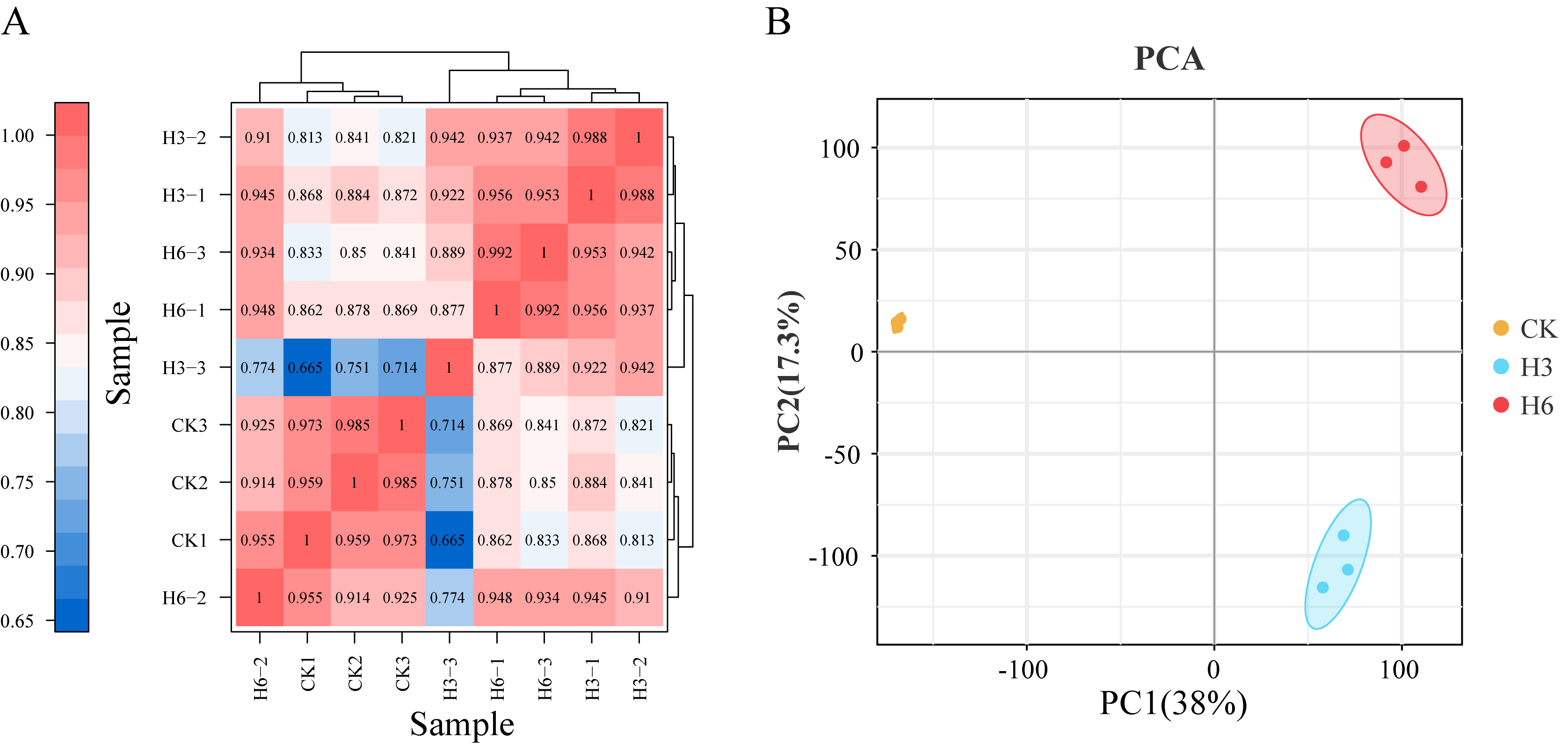

Supplement: Supplementary file 1 [file life-15-00697-s001.zip › Fig S1.tif]
